# Differential effects of *Apoe Christchurch (R136S)* mutation on amyloid plaque and tau pathology in transgenic mouse models

**DOI:** 10.1002/alz.091903

**Published:** 2025-01-03

**Authors:** Kristine M Tran, Shimako Kawauchi, Nellie Kwang, Cassandra Mar, Donna Chao, Celia Da Cunha, Andrea J Tenner, Frank M LaFerla, Grant R MacGregor, Kim N. Green

**Affiliations:** ^1^ Univ. of California Irvine, Neurobio. and Behavior, Irvine, CA USA; ^2^ University of California, Irvine, Irvine, CA USA; ^3^ Institute for Memory Impairments and Neurological Disorders (MIND), Irvine, CA USA; ^4^ Department of Neurobiology and Behavior, University of California, Irvine, CA USA

## Abstract

**Background:**

A case study on a PSEN1 (E280A) carrier with *APOECh* (R136S) mutation revealed changes in APOE protein function led to a protective effect on AD outcomes. Notably, there is an intriguing disparity between the two hallmark pathologies: a reduction in tauopathy but no change in plaque burden. Given that the APOE protein is predominantly produced by astrocytes and activated microglia, and the APOE gene is among the disease‐associated microglia (DAM) genes, it is conceivable that the variance in pathological outcomes may be rooted in the glial response. Therefore, studying the *APOEch* mutation will provide a unique opportunity to investigate the role of APOE in tau and Aβ plaque development, with a particular emphasis on the proposed link: neuroinflammation.

**Method:**

We used CRIPSR‐Cas9 to introduce *ApoeCh* into the mouse genome. Mice were then bred and crossed to homozygosity with the 5xFAD and PS19 transgenic mouse models to study the effect of the variant on amyloid plaque and tauopathy, respectively. Brain slices were immunolabeled for microglia and reactive astrocytes, and single‐cell spatial transcriptomics/proteomics was investigated using multiplexed error‐robust fluorescence in situ (MERFISH) technology. Plasma neurofilament light chain (NfL) along with Ab and tau protein levels were measured via MSD technology.

**Result:**

In the 5xFAD mouse model, analysis of confocal images reveals reduction in plaque load in the 5xFAD mice at 12 months of age that is reflected in the global decrease in microglia and astrocyte number with *ApoeCh*. However, there is an increase in DAM response to plaque observed through elevated CD11c^+^ staining along with upregulation of DAM genes and protein levels via spatial transcriptomics and proteomics. In the PS19 mice, although there is an increase in phospho‐tau level, there is a stark reduction in microgliosis to tauopathy. Plasma neurofilament light chain levels reveal that *ApoeCh* reduces plasma NfL level in response to plaques but not tau.

**Conclusion:**

*ApoeCh* mutation has differential effects on glial responses to plaque and tau. *ApoeCh* decreases plaque load and plasma NfL but increases inflammatory response to plaques. Whereas in a tau model, *ApoeCh* increases phosphorylated tau level but decreases microgliosis and inflammatory response to tau pathology.

